# Real‐Time Monitoring of Enzyme‐Catalysed Reactions using Deep UV Resonance Raman Spectroscopy

**DOI:** 10.1002/chem.201701388

**Published:** 2017-05-02

**Authors:** Chloe Westley, Heidi Fisk, Yun Xu, Katherine A. Hollywood, Andrew J. Carnell, Jason Micklefield, Nicholas J. Turner, Royston Goodacre

**Affiliations:** ^1^ School of Chemistry, Manchester Institute of Biotechnology University of Manchester 131 Princess street Manchester M1 7DN UK; ^2^ Department of Chemistry University of Liverpool Liverpool L69 7ZD UK

**Keywords:** biotransformations, on-line monitoring, quantification, reaction monitoring, UV resonance Raman (UVRR) spectroscopy

## Abstract

For enzyme‐catalysed biotransformations, continuous *in situ* detection methods minimise the need for sample manipulation, ultimately leading to more accurate real‐time kinetic determinations of substrate(s) and product(s). We have established for the first time an on‐line, real‐time quantitative approach to monitor simultaneously multiple biotransformations based on UV resonance Raman (UVRR) spectroscopy. To exemplify the generality and versatility of this approach, multiple substrates and enzyme systems were used involving nitrile hydratase (NHase) and xanthine oxidase (XO), both of which are of industrial and biological significance, and incorporate multistep enzymatic conversions. Multivariate data analysis of the UVRR spectra, involving multivariate curve resolution‐alternating least squares (MCR‐ALS), was employed to effect absolute quantification of substrate(s) and product(s); repeated benchmarking of UVRR combined with MCR‐ALS by HPLC confirmed excellent reproducibility.

Reaction monitoring based on analytical spectroscopy is broadly used to observe chemical changes in a variety of applications, including energy and fuel industries, bio‐based technologies and processes, pharmaceuticals, as well as for biocatalyst discovery and optimization.^1^ Reaction monitoring provides essential information in terms of molecular speciation, and affords key insights into reaction mechanisms, kinetics and the biochemical process of the system investigated. Furthermore, real‐time (in contrast to off‐line) reaction monitoring greatly improves the efficiency and accuracy of the overall process, with label‐free spectroscopic‐based methodologies being employed.^2^ Laborious sample preparation methods and purification steps are no longer required prior to analysis, thus minimising the need for transfers and sample handling, ultimately reducing errors. Advancements in engineering, such as the incorporation of robotics and sophisticated computational programs, lead to overall improvements and as a consequence, there is a significant reduction in the time taken for analysis.1c, 1d, 3

However, for biocatalytic applications, real‐time reaction monitoring provides specific challenges: the sensitivity required to monitor conversions is often an issue, because low substrate concentrations are commonly used.^4^ As a result, monitoring conversions involving detection/presence of intermediates in multi‐step biotransformations can be problematic. The most common method of measuring the rate of substrate turnover is the use of spectrophotometric assays.^5^ Although these assays are easy to use and interpret, a major limiting factor is the requirement for a fluoro‐/chromo‐genic reporter. However, in most cases, this means that the activity of the enzyme is detected indirectly or that improved enzyme activities may be selected based on the use of an idealised substrate, which may not translate to the real one. Whilst other spectroscopic and spectrometric physicochemical techniques are commonly employed (*viz*., NMR, HPLC and LC‐MS), these methods too have notable drawbacks, such as extensive sample preparation, high equipment costs, large solvent volumes, long acquisition times, and in some instances provide limited structural information.5a, 5b, 6 Therefore, there is a need for rapid, robust and reagent‐free on‐line high‐throughput screening methods to overcome these significant drawbacks.

Raman spectroscopy presents itself as an ideal analytical technique to use for screening applications, because it is rapid, non‐destructive and non‐invasive. Moreover, it can be performed *in situ* in aqueous environments and provides molecular specific information. We have previously shown that the conversion of glucose to ethanol by yeast can be monitored by Raman spectroscopy with a NIR excitation wavelength.^7^ However, Raman scattering is a relatively weak physical phenomenon and is often further exacerbated by fluorescence interference when excitation involves lasers in the visible EM.^8^ As a consequence, enhancement techniques are regularly employed to increase scattering efficiency. Surface‐enhanced Raman scattering (SERS), a surface‐sensitive Raman enhancement technique, has previously been used to monitor enzymatic biotransformations indirectly.^9^ Very recently, we successfully demonstrated a >30‐fold reduction in acquisition times for multiple enzymatic steps measuring analytes directly. This delivered high levels of accuracy and reproducibility, highlighting its suitability as an alternative screening technique.^10^ However, SERS requires a roughened metal surface that cannot be readily used for on‐line assessment of enzymatic reactions, so at best is only suitable for at‐line analysis.

Ultraviolet resonance Raman (UVRR) spectroscopy is a variant of “normal” Raman and involves the enhancement of Raman scattering by UV (in this case, at 244 nm). When the frequency of the laser coincides/matches the frequency of the molecule's electronic transition, enhancements of 10^3^–10^5^ can be observed.^11^ UVRR is an attractive technique for use in screening applications as the biotransformation(s) can be performed in real time; with no interference from background fluorescence (there is no fluorescence below 260 nm excitation).^12^ Moreover, the ability to measure analytes of interest directly without the need to quench the system, or have additional reagents as needed for SERS, is advantageous. Although this technique requires the absorption of laser light by chromophores in the UV region (most notably, from aromatics and fused ring systems), many complex biological systems fulfil this requirement, with nucleic acids and amino acids being particularly amenable to UVRR.^13^

Herein, we demonstrate how UVRR can be used for real‐time reaction monitoring using two different biocatalytic reactions (Figure 1). First, we focus on the conversion of nitriles to their corresponding amides using nitrile hydratase (NHase) (Scheme 1 a), a class of enzyme extensively used in chemical synthesis within various industries—with acrylamide, nicotinamide (vitamin B_3_) and pyrazinamide (anti‐tuberculosis agent) being notable examples.^14^ Second, to illustrate multiple reaction steps, we have applied the method to xanthine oxidase (XO) catalysed biotransformations (Scheme 1 b). XO catalyses the oxidation of a wide range of substrates including purines and xenobiotic compounds, with xanthine and hypoxanthine, its natural substrates, being the focus in this investigation.^15^

**Figure 1:**
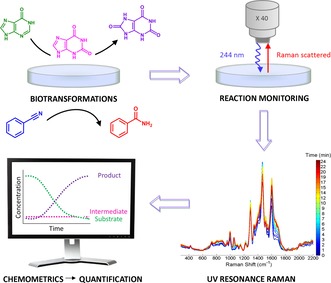
Workflow of the UVRR approach for real‐time reaction monitoring of multiple biotransformations.

**Scheme 1:**
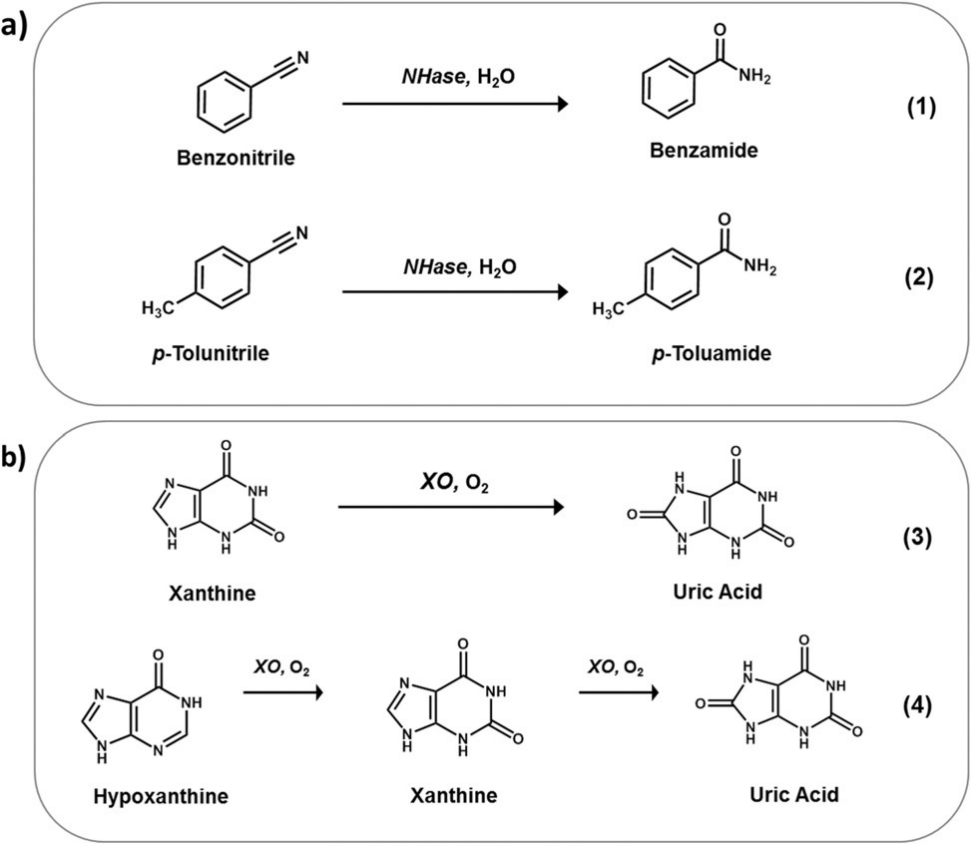
Biotransformations (1–4) selected for monitoring by UVRR: (a) bioconversions of nitriles to the corresponding amides by nitrile hydratase (NHase); (b) oxidation of purines by xanthine oxidase (XO).

The biotransformations of interest (1–4) are shown in Scheme 1. Reaction conditions for all biotransformations were optimised accordingly for UVRR monitoring (see methods in the Supporting Information for full details). For optimum UVRR spectra, a 20 second acquisition time and approximately 0.2 mW laser power at sample was required using an excitation wavelength in the deep UV at 244 nm. Characteristic UVRR spectra for each analyte, with unique peaks identified are summarised in Figure 2 (see Tables S1 and S2 in the Supporting Information for tentative band assignments). To monitor the enzyme‐catalysed biotransformations, the instrument had to be modified and optimised (see Figure S1 in the Supporting Information); briefly, a magnetic stirrer plate was inserted below the turntable, with the reaction vessel (containing a magnetic stirrer bar) on top, focused under the microscope objective. The reaction was initiated upon the introduction of enzyme. Continuous stirring permitted maximal enzyme–substrate interaction throughout the reaction and provides a true representation of the conversion of substrate(s) to product(s). This set‐up also allows the energy from the laser source to be evenly distributed over a much larger volume. Furthermore, to minimise the risk of reduced focus on the sample through solvent evaporation and removal of volume for HPLC analysis, the reaction was performed on a 10 mL scale. An initial concern was the integrity of the sample when subjected to a highly powered laser; however, no spectral changes (and hence, no photo‐degradation) was observed throughout the reaction time course (see the Supporting Information, “Photo‐degradation of sample” section and Figure S2). Interestingly, from these investigations, we observed bathochromic shifts (as a function of pH) for XO analytes (see the Supporting Information, section “Bathochromic shifts of XO analytes” and Figures S3–S5). Although there are characteristic peaks for each analyte, thus distinguishing starting material from product, the UVRR spectra were highly similar with many overlapping peaks (especially for XO analytes). Therefore, for all biotransformations, multivariate curve resolution‐alternating least squares (MCR‐ALS) was employed. MCR‐ALS is a popular feature extraction tool for mixture analysis and was used to extract the necessary information (pure component spectra and corresponding concentrations) to predict absolute levels of the analytes within a mixture (see the Supporting Information, Figure S6 for a flow diagram summarising this MCR‐ALS approach).^16^

**Figure 2:**
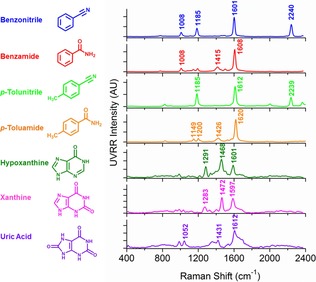
Average UVRR spectra (*n=*5) of each analyte for both biotransformations: benzonitrile (blue), benzamide (red), *p*‐tolunitrile (bright green) and *p*‐toluamide (orange), hypoxanthine (green), xanthine (pink) and uric acid (purple). For NHase analytes, spectra were obtained at 12.5 mm, pH 7.2. For XO analytes, spectra were obtained at 0.75 mm, pH 7.6. All spectra are representative of starting reaction concentrations with characteristic peaks annotated. UVRR spectra were obtained for 20 s with baseline correction, normalisation and smoothing applied (see the Supporting Information, “Data processing” for full details).

We initially looked at the conversion of benzonitrile to benzamide (biotransformation 1) with <50 % conversion achieved over a 20 minute time period. The deconvolved spectra for each analyte were highly similar to the UVRR spectra from the pure substrate and product (see the Supporting Information, Figure S7). For brevity purposes, we only represent the graphical results of one replicate. Figure 3 a shows the UVRR spectra over the reaction time course, illustrating (by use of a colour bar) the increase and decrease of characteristic peaks with respect to time. Time points with both HPLC and UVRR data were used as the training set for the MCR‐ALS model (i.e., HPLC was used as external validation—see SI Figure S8 for HPLC calibration). Time points with UVRR data (but without HPLC data) were used as the test set. As can be observed from Figure 3 b, the UVRR predictions are in excellent agreement with the HPLC results, which is reflected by high *R* ^*2*^ values across all replicates, with an average of 0.964 and 0.983 for substrate and product, respectively (see Table 1). The coefficient of determination, *R* ^*2*^, is the proportion of variability in a data set that is accounted for by a statistical model (in this case MCR‐ALS) with *R* ^*2*^ values closer to one indicating an excellent fit. Notably, this experiment was conducted on five separate occasions, over a four‐week period thus accounting for day‐to‐day instrument variance, ultimately demonstrating its robustness for on‐line reaction monitoring.

**Figure 3:**
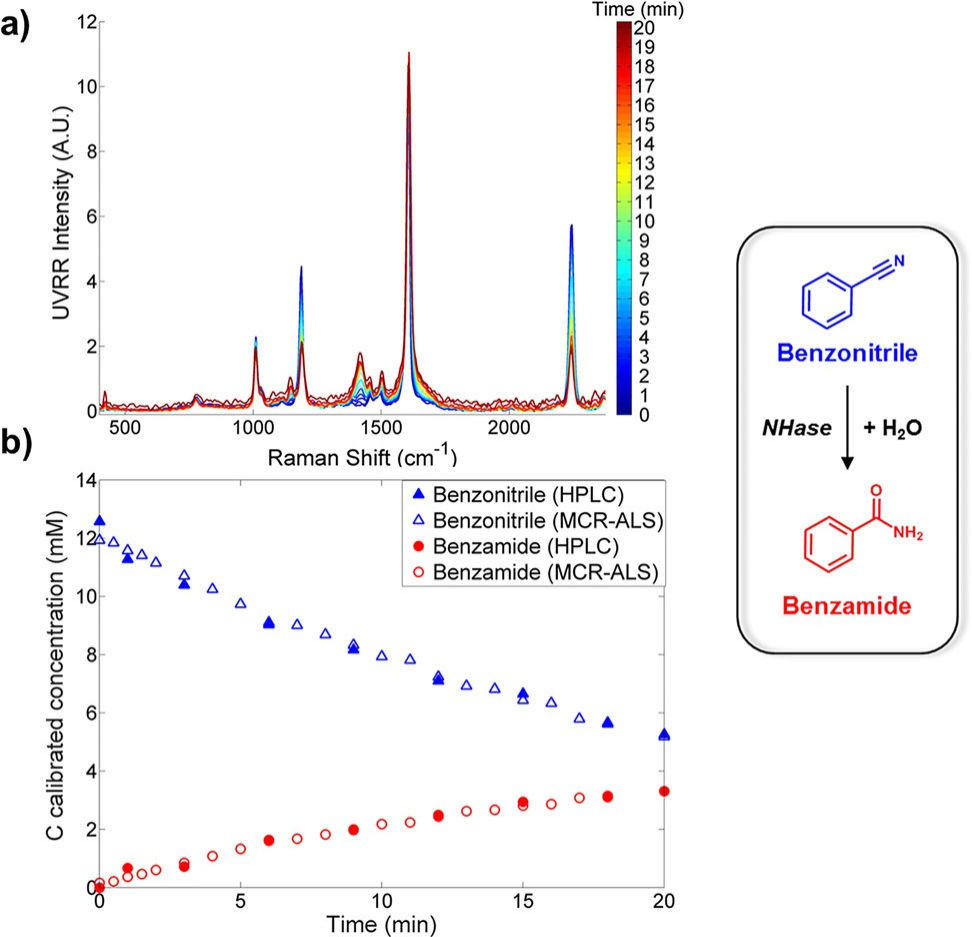
MCR‐ALS model was applied to the UVRR data for the conver‐ sion of benzonitrile to benzamide (biotransformation 1). a) Accumulative spectra taken over the 20 min time course. The colour bar highlights each time point monitored with the start (*t=*0) in blue and the end point (*t=*20) in red. b) Reaction dynamics from real‐time UVRR measurements (denoted by outlined symbols) and off‐line HPLC data (denoted by solid symbols) as a function of time. UVRR spectra were obtained for 20 s with baseline correction, normalisation and smoothing applied (see the Supporting Information “Data processing” for full details). Data shown from replicate 2.

**Table 1:** Summary of the regression coefficients (*R* ^*2*^) across all five replicates for the two separate biotransformations.^[a]^

Replicate	NHase (biotransformation 1)		XO (biotransformation 3)	
	Benzonitrile *R* ^2^	Benzamide *R* ^2^	Xanthine *R* ^2^	Uric acid *R* ^2^
1	0.959	0.993	0.990	0.955
2	0.987	0.987	0.954	0.977
3	0.969	0.982	0.916	0.965
4	0.962	0.980	0.973	0.987
5	0.942	0.973	0.957	0.983

[a] Biotransformation 1 (benzonitrile to benzamide) using NHase, with overall mean *R* ^*2*^ values of 0.964 and 0.983, respectively. Biotransformation 3 (xanthine to uric acid) using XO, with overall mean *R* ^*2*^ values of 0.958 and 0.973, respectively. These high *R* ^*2*^ (that are close to 1) indicate excellent fit.

Moreover, to extend this approach, we next investigated a similar NHase substrate, *p*‐tolunitrile—only differing by a CH_3_ group, yet possessing unique peaks compared to benzonitrile. Once again, with this biotransformation (biotransformation 2), we were able to monitor the reaction successfully. The UVRR and HPLC results were in very good agreement with one another, with typical *R* ^2^ values of 0.898 and 0.914 for *p*‐tolunitrile and *p*‐toluamide, respectively (see the Supporting Information, Figure S9 and Table S3).

To demonstrate versatility of UVRR combined with MCR‐ALS, we then performed analysis on a different, second enzyme system: XO (biotransformations 3 and 4). We have previously shown that we can monitor these conversions using SERS, and as has been already discussed, this involves the use of additional reagents preventing real‐time monitoring.^10^ Therefore, this UVRR approach should overcome this main drawback. Furthermore, this enzyme system in itself provided a challenge with the analytes being highly similar in structure, only differing by additional carbonyl groups (Scheme 1 b). First, the two‐analyte conversion (biotransformation 3) of xanthine to uric acid was investigated, with >50 % conversion achieved in 18 minutes. Adopting the same process, the MCR‐ALS model was applied to the reaction data with results being in excellent agreement with the HPLC analysis (see Figure 4 and the Supporting Information, Figure S10 for deconvolved spectra of each analyte and Figure S11 for HPLC calibration). Average *R* ^2^ values of 0.958 and 0.973 were obtained for xanthine and uric acid, respectively (Table 1). We then extended this to a third analyte to include the precursor hypoxanthine (biotransformation 4), ultimately demonstrating the flexibility of this real time, on‐line reaction monitoring screen for a more complex, multicomponent reaction system. The reaction conditions were modified slightly, with <40 % conversion reached after 35 minutes. Again, MCR‐ALS analysis was employed with the deconvolved UVRR spectra being highly consistent with the pure spectra for each analyte (Figure 5 a–c). The UVRR predictions were in very good agreement with the HPLC results (see Figure 5 d). The *R* ^2^ values were slightly lower than biotransformation 3 (see the Supporting Information, Table S4), which was to be expected due to the increased complexity of the system, as well as the highly similar spectra between the three analytes. Noticeably, the *R* ^*2*^ value of xanthine was lower (biotransformation 4) than previous—this is due to the low overall concentration of xanthine (<8 %) throughout the reaction. This was further supported by the proposed mechanism of XO (based on xanthine dehydrogenase, XDH, from *Rhodobacter capsulatus*), in which hypoxanthine binds to the active site and is converted to xanthine by oxidation at the C‐2 position. Xanthine is then released, before binding in a different orientation to present the C‐8 for oxidation to give uric acid.^17^ This means that the concentration of the intermediate remains low throughout.

**Figure 4:**
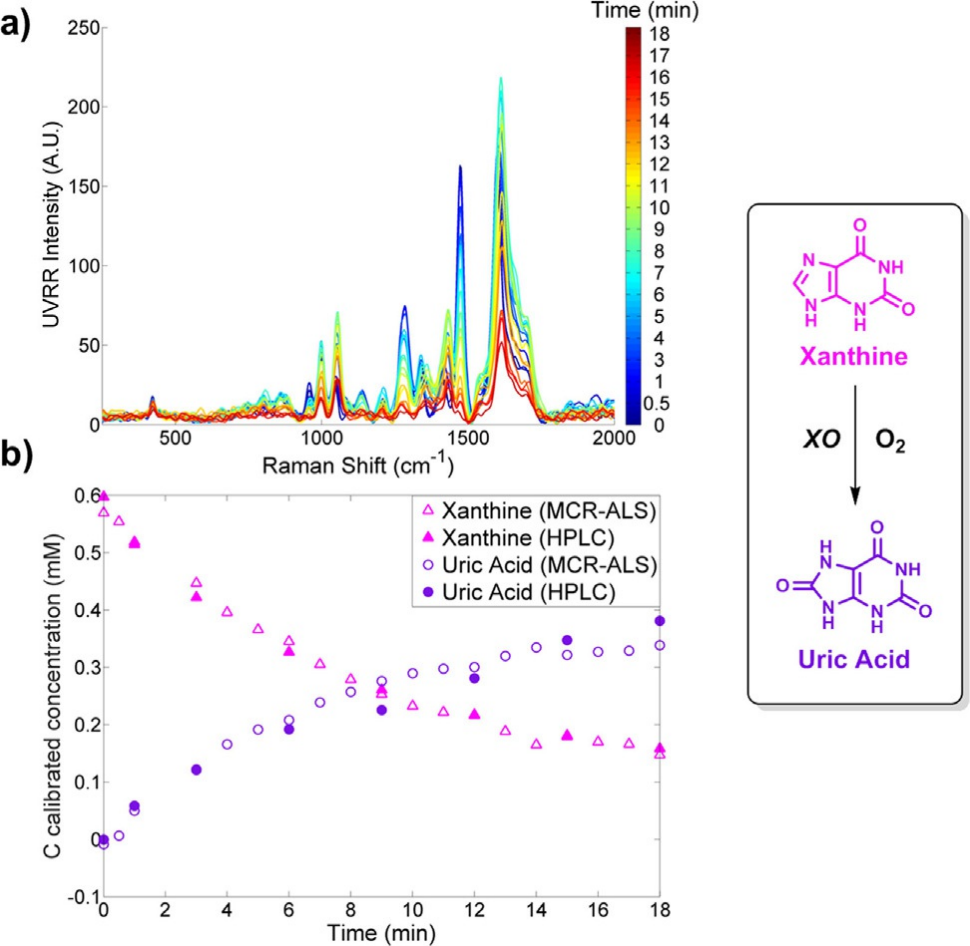
MCR‐ALS model was applied to the UVRR data for the conver‐ sion of xanthine to uric acid, biotransformation 3. a) Accumulative spectra taken over the 18 min time course. The colour bar highlights each time point monitored with the start (*t=*0) in blue and the end point (*t=*18) in red. b) Reaction dynamics from real‐time UVRR measurements (denoted by outlined symbols) and off‐line HPLC data (denoted by solid symbols) as a function of time. UVRR spectra were obtained for 20 s with baseline correction, normalisation and smoothing applied (see the Supporting Information “Data processing” for full details). Data shown from replicate 1.

**Figure 5:**
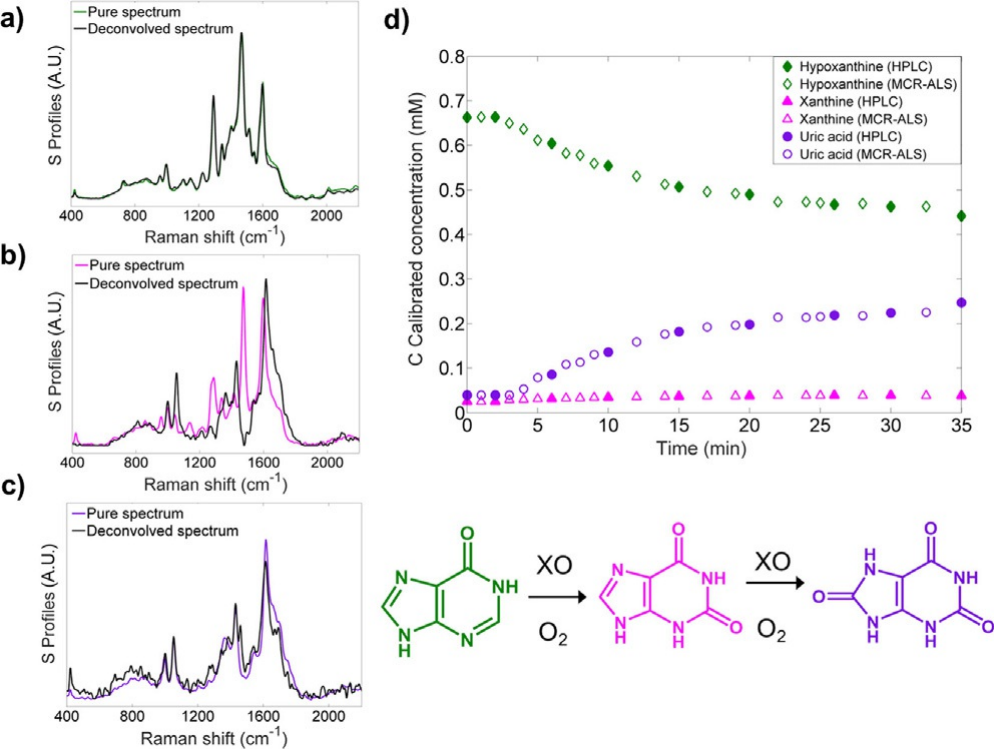
MCR‐ALS model was applied to the UVRR data, in which it successfully deconvolved spectra into its pure components for biotransformation 4: a) hypoxanthine; b) xanthine; and c) uric acid. d) Reaction dynamics from real‐time UVRR measurements (denoted by outlined symbols) and off‐line HPLC data (denoted by solid symbols) as a function of time for the conversion of hypoxanthine to xanthine to uric acid. UVRR spectra were obtained for 20 s with baseline correction, normalisation and smoothing applied (see the Supporting Information “Data processing” for full details). Data shown from replicate 1.

Where this work could be further explored includes investigating the two separate pathways known to catalyse the conversion of nitrile containing compounds into their corresponding carboxylic acid: either in a single step (nitrilase) or a two‐step process (nitrile hydratase and amidase; see the Supporting Information, Figure S12 a). Fluorometric and colorimetric assays have previously been reported, including successful differentiation between the two pathways; however, only semi‐quantitative analysis has been possible.^18^ Extending on biotransformation 1, we have shown that we can potentially use this UVRR approach to monitor such cascades as the corresponding carboxylic acid involved in this pathway has unique, characteristic peaks (see the Supporting Information, Figure S12 b). Furthermore, other nitrile containing substrates, for example, 3‐pyridinecarbonitrile and pyrazinecarbonitrile, which are precursors for important pharmaceutical products, can similarly be monitored (see the Supporting Information, Figure S12 c and S12 d). These results further demonstrate the general utility of the UVRR approach for enzyme reaction monitoring.

In this study, we have developed a label‐free, rapid, on‐line screening method to monitor biological and industrially relevant biotransformations based on UVRR spectroscopy. To demonstrate the general utility of this approach, multiple substrates and enzyme systems were investigated, which included single, multiple and cascade enzyme systems. UVRR spectra acquisitions were rapid (20 s per measurement) and when combined with MCR‐ALS produced substrate(s) and product(s) concentrations that were completely in agreement with off‐line HPLC measurements. Additional benchmarking involved repeat biotransformations conducted over several weeks and this established the excellent reproducibility and robustness of this new analytical approach. In conclusion, we believe that additional optimisation and configuration of the UVRR instrument set‐up will make this approach amenable to miniaturization and *in situ* point‐and‐shoot analyses,^19^ thus enhancing the potential for wider application. The method could also be developed as a high‐throughput screening technique for enzyme activity, including the monitoring of cascade biotransformations, as well as for investigating enzyme inhibitors.

## Conflict of interest

The authors declare no conflict of interest

## Supporting information

As a service to our authors and readers, this journal provides supporting information supplied by the authors. Such materials are peer reviewed and may be re‐organized for online delivery, but are not copy‐edited or typeset. Technical support issues arising from supporting information (other than missing files) should be addressed to the authors.

Supplementary
