# Supplementary material for: Real‐Time Monitoring of Enzyme‐Catalysed Reactions using Deep UV Resonance Raman Spectroscopy
Source: Chemistry. 2017 May 2;23(29):6983–7. doi: 10.1002/chem.201701388 (PMC5488198; doi:10.1002/chem.201701388)
Supplement: Supplementary file 1 — Supplementary [file CHEM-23-6983-s001.pdf]

CHEMISTRY
A European Journal

Supporting Information

Real-Time Monitoring of Enzyme-Catalysed Reactions using Deep UV Resonance Raman Spectroscopy

Chloe Westley+,[a] Heidi Fisk+,[a] Yun Xu,[a] Katherine A. Hollywood,[a] Andrew J. Carnell,[b] Jason Micklefield,[a] Nicholas J. Turner,[a] and Royston Goodacre*[a]

chem_201701388_sm_miscellaneous_information.pdf

Reagents and materials. All chemical reagents were of analytical grade and used with no additional purification unless otherwise stated. Acetic acid (HPLC), acetonitrile (HPLC), benzonitrile (≥99%), benzamide (≥99%), benzoic acid (≥99%), butanoic acid (≥99%), hypoxanthine (≥99%), methanol (HPLC), nicotinamide (≥98.5%), nicotinic acid (≥99.5%), 3-pyridinecarbonitrile (≥98%), pyrazinecarbonitrile (≥99%), *p*-tolunitrile (≥98%), *p*-toluamide (≥99%), sodium acetate trihydrate (≥99%), uric acid (≥99%), water (HPLC), xanthine (≥99%), xanthine oxidase microbial lyophilized powder (EC 1.17.3.2) were purchased from Sigma Aldrich Ltd. (Dorset, UK). Potassium dihydrogen phosphate, dipotassium phosphate, pyrazinamide (≥99%), sodium carbonate, and sodium hydroxide were obtained from Fischer Scientific (Loughborough, UK). Nitrile hydratase was purchased from Prozomix (PRO-NHASE (018), LOT 2013-1, EC 4.2.1.84) (Northumberland, UK).

Supplementary methods

Reaction conditions for biotransformations 1 and 2. A stock solution of nitrile hydratase (as sold by Prozomix) was prepared as follows: 250 µL nitrile hydratase solution was pelleted by centrifugation (1 min, 21,000 *g*) and the supernatant removed. The pellet was then re-suspended in 2 mL of TRIS buffer (4.0 x 10^{-2} M) and butanoic acid (4.0 x 10^{-2} M) at pH 7.2 before use (stock concentration of enzyme not known). Starting reaction mixture contained either benzonitrile (biotransformation 1) or *p*-tolunitrile (biotransformation 2) dissolved in potassium phosphate buffer at pH 7.2 (final concentration 1.25 x 10^{-2} M with 2.5% MeOH) and nitrile hydratase solution (40 µL for biotransformation 1, 200 µL for biotransformation 2).

Reaction conditions for biotransformations 3 and 4. A stock solution of xanthine oxidase was prepared by dissolving 7 mg of xanthine oxidase microbial lyophilized powder in 1.1 mL potassium phosphate buffer at pH 7.6 (stock concentration 2.25 × 10^{-5} M). Starting reaction mixture contained either xanthine (biotransformation 3) or hypoxanthine (biotransformation 4) dissolved in water (final concentration 7.5 × 10^{-4} M), potassium phosphate buffer at pH 7.6 (final concentration 0.3 M) and xanthine oxidase (120 µL for biotransformation 3, 150 µL for biotransformation 4).

Reaction sample preparation and monitoring

For all biotransformations, the reaction mixture was focused under the microscope objective in a small, glass Petri dish (see Figure 3.5). At various time points after enzyme addition, UVRR and HPLC spectra were collected throughout the biotransformation. To minimise the risk of reduced focus on the sample through solvent evaporation and removal of volume (for HPLC analysis), the reaction was performed on a 10 mL scale.

For biotransformation 1 and 2, at specific time points, 20 µL of sample was removed from the reaction mixture and immediately quenched and diluted with 180 µL with MeOH. The sample was then centrifuged at 21,000 ×*g* for 6 min. 100 µL of the sample was then transferred to a HPLC vial and subjected to HPLC analysis.

For biotransformations 3 and 4, at specific time points, 20 µL of sample was removed from the reaction mixture and immediately diluted to 80 µL with water. The sample was then

heated for 5 min at 80°C and centrifuged at 14000 ×*g* for 4 min. 60 µL of the sample was then transferred to a HPLC vial and subjected to HPLC analysis.

Instrumentation

UV-Vis absorption analysis was carried out using Thermo Biomate 5 (Thermo Fisher Scientific Inc., Massachusetts, USA). 1 mL of the sample was pipetted into a quartz cuvette and inserted into a sample holder. Data were acquired over a wavelength range of 210-350 nm.

HPLC Analysis for biotransformations 1 and 2. HPLC separation was conducted using an Agilent Zorbax Eclipse Plus HPLC system set up for reverse phase separation consisting of a diode array detector. For both biotransformations, the column was a 100 × 4.6 mm, Phenomenex Eclipse Plus® C18 with a 3.5 µm particle size. For each injection, the run time was 12.0 min pumped at a flow rate of 1 mL min^{-1} and at 30°C column temperature. The mobile phase consisted of a linear gradient, starting conditions of 5% MeCN/H_2O (plus 0.05 % TFA) held for 2 min before increasing to 75 % MeCN/H_2O over 6 min. Prior to washing at 95 % MeCN/H_2O for 1.5 min and re-equilibration to initial conditions over 2.5 min (total run time 12 min). 5 µL of each sample was introduced using an auto-injector. UV absorbance was detected at 254 nm throughout.

HPLC Analysis for biotransformations 3 and 4. HPLC separation was conducted using an Agilent 1100 series HPLC system set up for reverse phase separation consisting of a diode array detector. For biotransformation 3, the column was 150 × 4.6 mm, Phenomenex Hyperclone C18 with a 5 µm particle size. For each injection, the run time was 10.0 min. The mobile phase was 0.020 M aqueous potassium phosphate buffer at pH 6.5, pumped at a flow rate of 1.0 mL min^{-1}. For biotransformation 4, the column was a 250 × 4.6 mm, ACE 5 C18-AR (Reading, Berkshire) with a 5 µm particle size. For each injection, the run time was 12.0 min. The mobile phase was 0.047 M aqueous acetic acid buffer at pH 4.65, pumped at a flow rate of 1.0 mL min^{-1}. For both biotransformations, 50 µL of each sample was introduced using an auto-injector. UV absorbance detection was measured at 254 nm.

UVRR analysis for all biotransformations. UVRR was performed using a Renishaw Raman 1000 system (Renishaw, Wotton-under-edge, Gloucestershire, UK). Approximately ~0.2 mW of power was delivered to the sampling point using a Lexel Model 95 ion laser emitting at 244 nm. The solution was continuously stirred under the laser to avoid photo-degradation using a magnetic stirrer plate and magnetic bar. Spectra were collected with an acquisition time of 20 s. Only spectra with no demonstrable photo-degradation and signal from the reaction vessel were used for analysis.

Data Processing

All data were exported from the respective instrument operating software and analysed using Matlab R2015a (The Mathworks, Natick, MA, USA).

HPLC data analysis

The peaks of the target analytes were integrated with the results of the HPLC data and served as an external validation data set to independently verify the accuracies of the MCR-ALS models in prediction.

UVRR data analysis

Multivariate curve resolution - alternating least squares (MCR-ALS) was employed due to the fact that the UVRR spectra of all three analytes are highly similar and do not possess characteristic peaks.[1] In MCR-ALS, the UVRR spectra were first baseline corrected, smoothed using wavelet smoothing and then normalised so that the sum of squares of each spectrum equals 1. This is to account for the decreasing signal strength and relatively increasing noise (attributed to the removal of aliquots for HPLC analysis (and specifically for biotransformation 1 and 2 the inherent volatility of the solution).

The spectra of all the monitored time points of a single reaction were then augmented to form a $t \times n$ data matrix X where t is the number of time points monitored and n is the number of wavenumbers recorded. Multivariate curve resolution using alternating least squares (MCR-ALS) was performed to deconvolve X into a product of two sub matrices C and S where C contains the "profiles" of the change in the concentrations the reactants during the reaction while S is the matrix storing the resolved spectra the reactants. Non-negativity constraint was applied to both concentration profile C and spectral profile S and each deconvolved pure spectrum had a unit norm (*i.e.* the sum of squares of each spectrum equals 1).
For the time points when HPLC measures were also taken, a linear regression model was built between the concentration profile of each reactant in C and the corresponding concentration measured by HPLC. The regression model was then applied to the whole concentration profile to get UVRR calibrated concentrations of the reactant over the whole monitored period of the reaction.

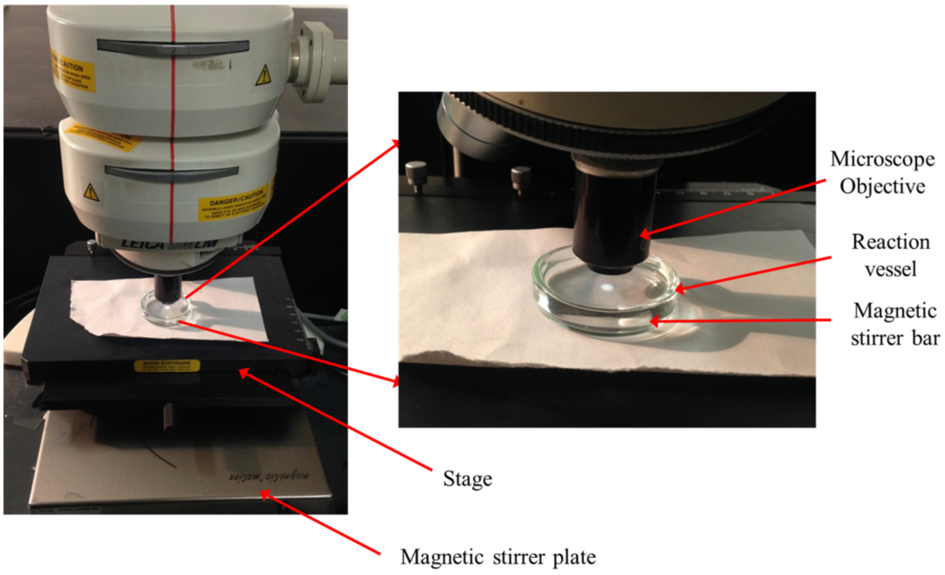

Figure S1 Annotated instrument set-up to monitor biotransformation using UV resonance Raman spectroscopy. The x40 UVRR objective was carefully focused onto the reaction mixture (10 mL scale), with 100% power on sample (~ 0.2mW at sampling point). Throughout the course of the reaction, samples for HPLC analysis were removed as well as UVRR data collected (20 s spectral acquisitions). The solution was constantly stirred using the magnetic stirrer plate beneath the stage and the magnetic stirrer bar in the reaction vessel.

Photo-degradation of sample:

We initially tried to photodegrade our sample so we were aware of the spectral changes to expect if the sample started to degrade or ‘burn’. Using biotransformation 3 as an example, we tried to photo-degrade the starting material, xanthine (in solution). After 30 min of constant interrogation of the laser on the sample, there were no changes in the spectra and thus no photodamage. Our only observation was the evaporation of solvent meaning as time increased, the sample point became out of focus and hence the spectra became nosier. We next looked at photo-degrading the corresponding solid sample, and after 45 min (vastly exceeding the total reaction time) we noticed broadening of peaks around 1550 cm^{-1} region (from C-C), similar to spectra of graphitic carbon-type species and charcoal, thus indicating ‘burning’ of the sample. These observations are in agreement with the literature.[2]

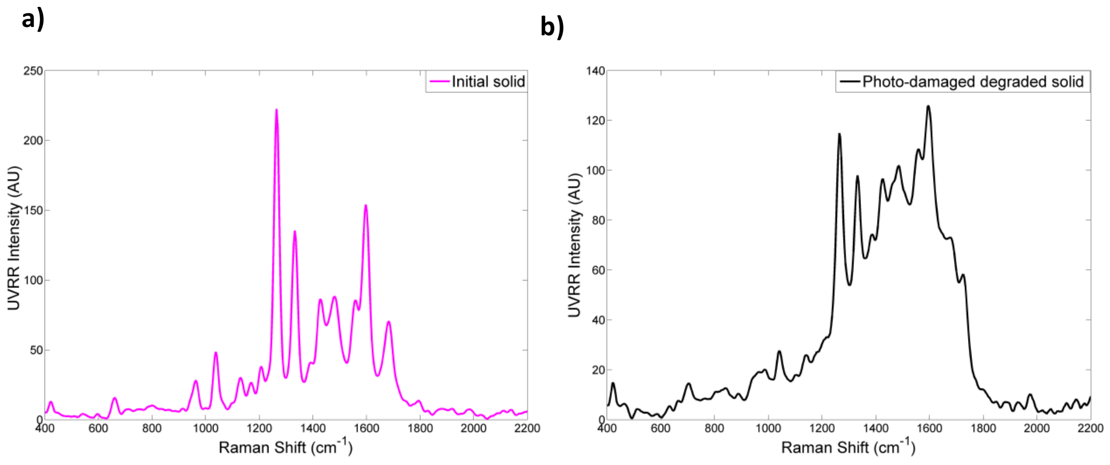

Figure S2. Average UVRR spectra (n=5) of a) initial solid xanthine sample (pink) and b) degraded solid xanthine sample (black) after 45 min (after each min, a 20 s spectral acquisition was acquired). A broadening of peaks around 1550 cm^{-1} indicates C-C presence suggesting photo-degradation.

Tables S1. Tentative UVRR band assignments for nitrile hydratase catalysed biotransformations at pH 7.2: benzonitrile and benzamide (biotransformation 1) and *p*-tolunitrile and *p*-toluamide (biotransformation 2)

**Raman Shift (cm^{-1})**				**Tentative band assignment**[3]
**Benzonitrile**	**Benzamide**	***p*-Tolunitrile**	***p*-Toluamide**	
		825 (w)	831 (w)	Ring breathing
1008 (m)	1008 (w)			C-C-C trigonal breathing
	1145 (vw)		1149 (m)	NH_2 rocking mode
1185 (m)	1192 (w)	1185 (s)	1200 (m)	C-H in-plane bend
	1415 (m)		1426 (m)	C-N stretch
1601 (vs)	1608 (vs)	1612 (vs)	1620 (vs)	C-C in-plane stretch
2240 (s)		2239 (s)		C≡N stretch

vs-very strong, s-strong, m-medium, w-weak, vw-very weak, br-broad, sh-shoulder

Table S2. Tentative UVRR band assignments for xanthine oxidase catalysed biotransformations at pH 7.6: xanthine to uric acid (biotransformation 3) and hypoxanthine to xanthine to uric acid (biotransformation 4)

Raman Shift (cm^{-1})			Tentative band assignment[4]
**Hypoxanthine**	**Xanthine**	**Uric Acid**	
1601 (s)	1597 (vs)	1612 (s)	C-N
		1540 (wsh)	-
1468 (vs)	1472 (vs)		C-N or C-C
1347 (m)	1410 (w)	1431 (m)	C-O
	1336 (s)	1359 (sh)	-
1291 (s)	1283 (s)		C-N, C-H bend
1223 (w)			Mixed ring vibrations, C-N
	1041 (w)	1052 (s)	Ring vibrations
995 (m)	995 (w)	995 (m)	N-H bend, ring trigonal deformation

vs-very strong, s-strong, m-medium, w-weak, vw-very weak, br-broad, sh-shoulder

Bathochromic shifts of XO analytes

Photo-degrading the sample led to an interesting observation regarding the UVRR spectra of xanthine. Notably, there were significant changes in spectral band positions and intensity between the solid spectra from xanthine and that in solution. Changes in environmental conditions (*e.g.* temperature, solvent, pH *etc.*) can lead to changes in some vibrational frequencies especially as for some molecules, these functional groups will interact differently with the solvent (through H-bonding, as well as acidic/basic ions). This led to a pH investigation looking at the associated UV-Vis absorption of xanthine and UVRR spectra, with the results indicating the observation of a bathochromic shift on increasing pH. In acidic medium (*i.e.* low pH), the nitrogen atom is free to lose its lone pair of electrons thus decreasing the delocalisation in the ring, leading to a decrease in conjugation, as observed in Figure 3.7, consequently, the molecule becomes less energetic. As the energy needed for excitation is higher, there is a shift to absorbing at shorter wavelengths. Conversely, in alkaline medium (*i.e.* high pH), the opposite phenomenon occurs, and the oxygens lone pairs increase delocalisation and conjugation to the ring, (as observed in Figure 3.7) meaning the molecule becomes more energetic, so less energy is required for excitation thus shifting to absorbing at longer wavelengths. This is supported by the increase in wavelength absorbance from λ_{max} 267 to 272 nm when going from pH 3.6 to 9.2. Figure 3.7 c and d) highlight key peaks that are affected by changing the pH of the solution. A similar, less pronounced effect was observed for hypoxanthine and uric acid (see Figure 3.8 and 3.9).[5]

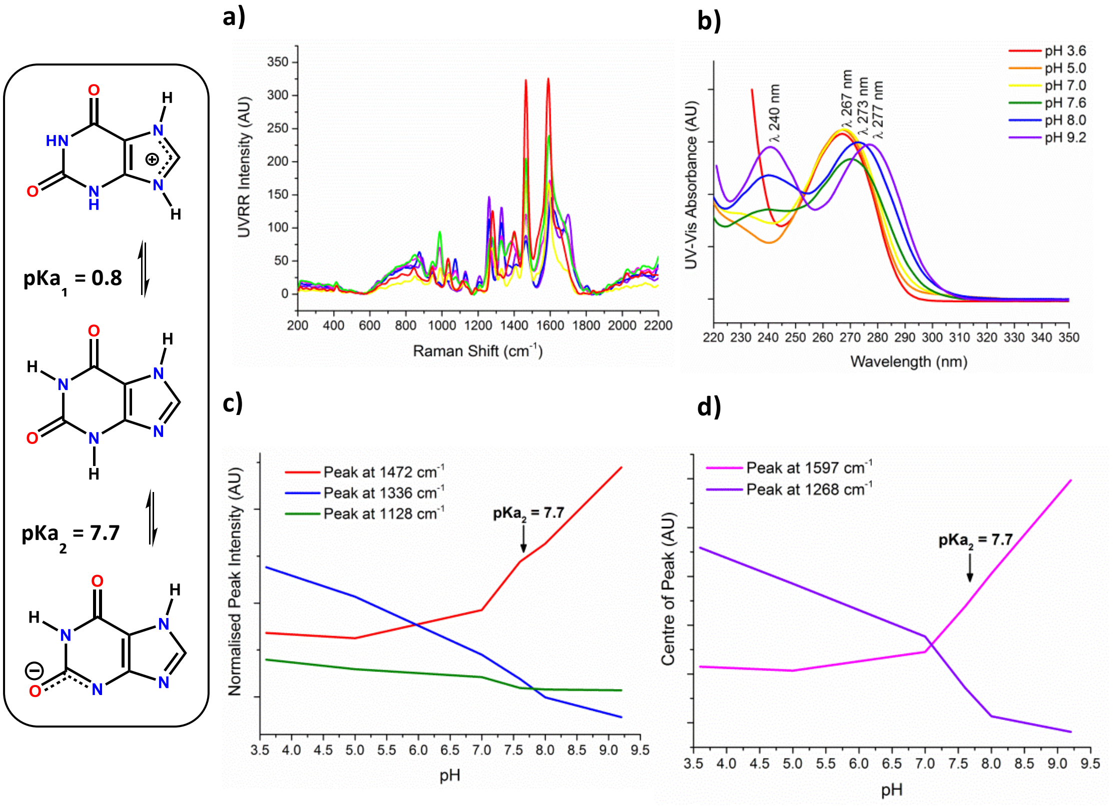

Figure S3. a) Average UVRR spectra (*n*=5) and b) UV-Vis absorbance spectra of xanthine at various pH: 3.6, 5.0, 7.0, 7.6, 8.0, and 9.2. A bathochromic shift in the UV-Vis absorption spectra was observed on increasing the pH from pH 3.6 to 9.2, consequently, the UVRR spectra of xanthine changed due to it being in different ionisation states. c) The intensity difference of the key peaks that change and d) a plot of the centre of the peaks that shift on increasing the pH of the solution from pH 3.6 to 9.2.

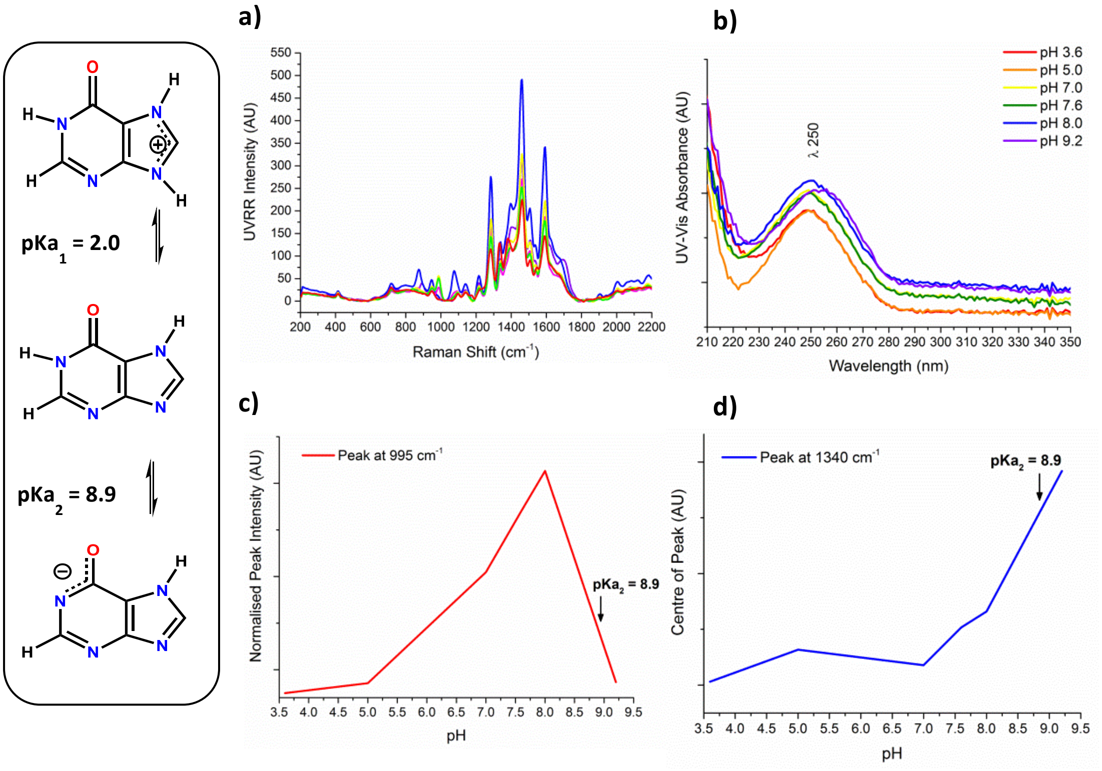

Figure S4. a) Average UVRR spectra (*n*=5) and b) UV-Vis absorbance spectra of hypoxanthine at various pH: 3.6, 5.0, 7.0, 7.6, 8.0, and 9.2. A bathochromic shift in the UV-Vis absorption spectra was observed on increasing the pH from pH 3.6 to 9.2, consequently, the UVRR spectra of hypoxanthine changed due to being in different ionisation states. c) The intensity difference of the key peak that changes and d) a plot of the centre of peak that shifts on increasing the pH of the solution from pH 3.6 to 9.2.

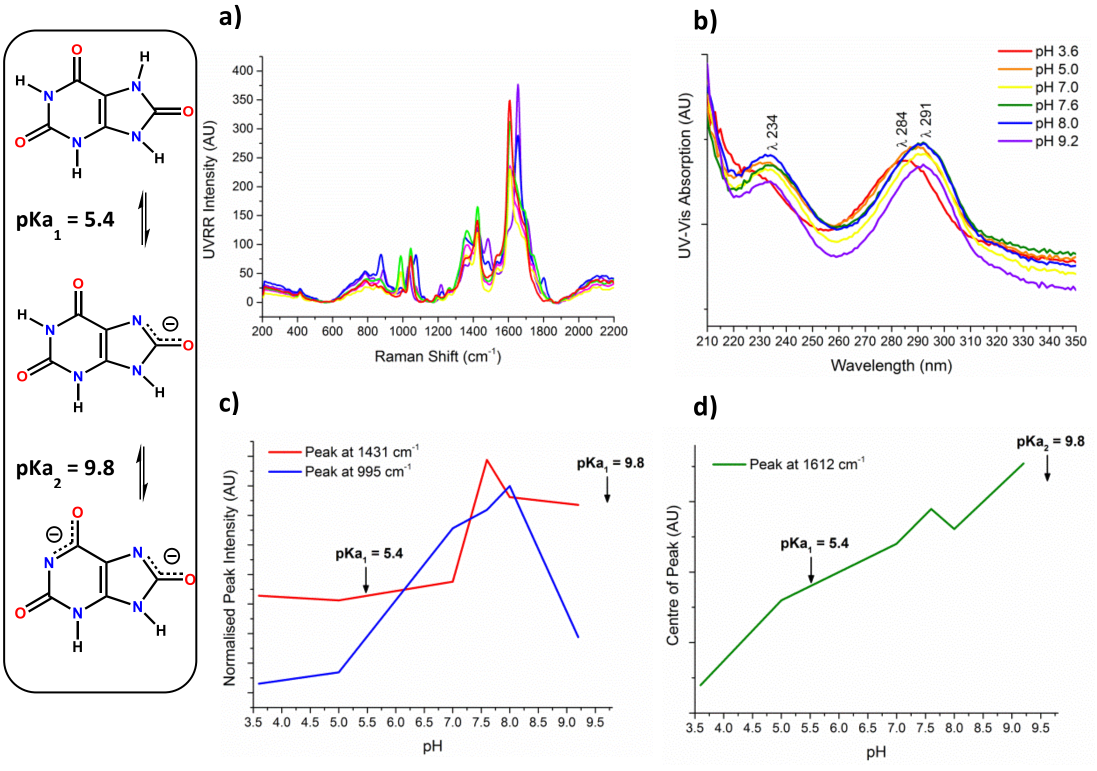

Figure S5. a) Average UVRR spectra (*n*=5) and b) UV-Vis absorbance spectra of uric acid at various pH: 3.6, 5.0, 7.0, 7.6, 8.0, and 9.2. A bathochromic shift in the UV-Vis absorption spectra was observed on increasing the pH from pH 3.6 to 9.2, consequently, the UVRR spectra of uric acid changed due to being in different ionisation states. c) The intensity difference of the key peaks that change and d) a plot of the centre of peak that shifts on increasing the pH of the solution from pH 3.6 to 9.2.

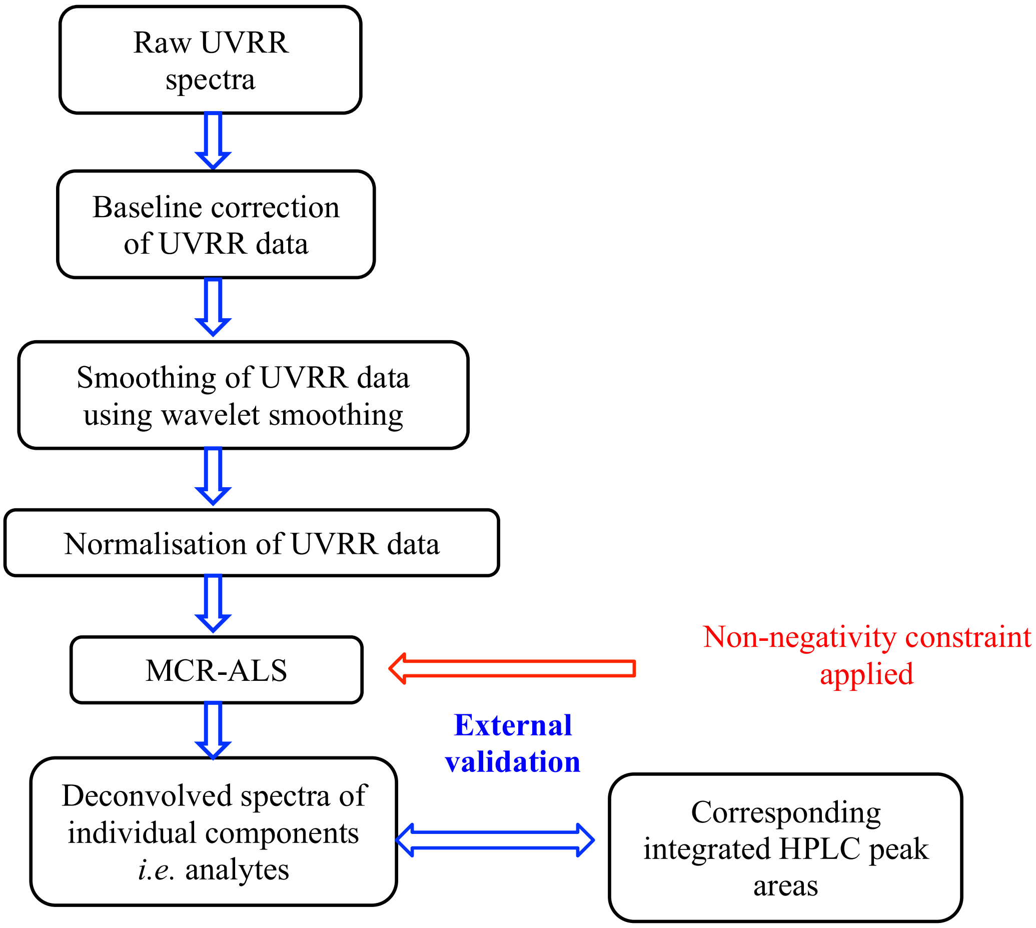

Figure S6. Flow diagram summarising the data pre-processing and MCR-ALS process used in predicting concentrations of each analyte from the reaction.

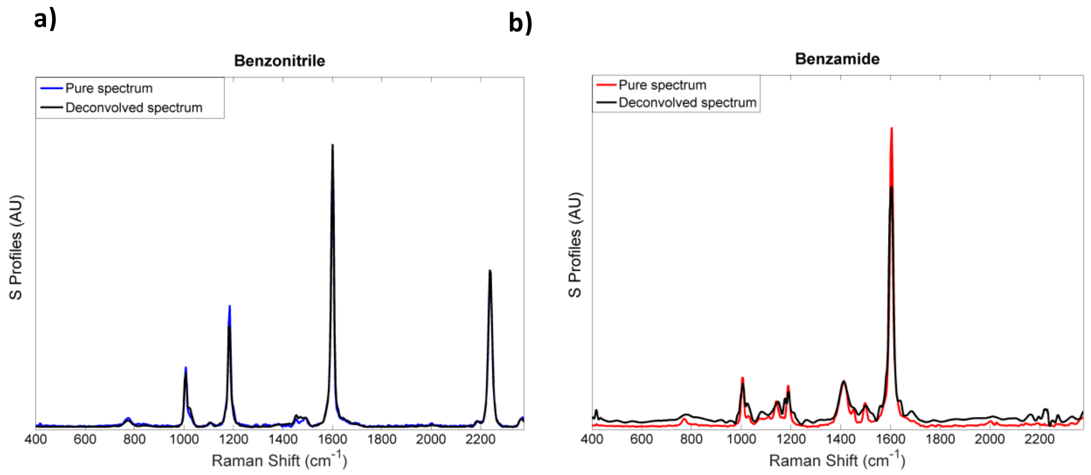

Figure S7. An MCR-ALS model was applied to the UVRR data where it successfully deconvolved spectra into its pure components a) benzonitrile (substrate) and b) benzamide (product) as shown for biotransformation 1.

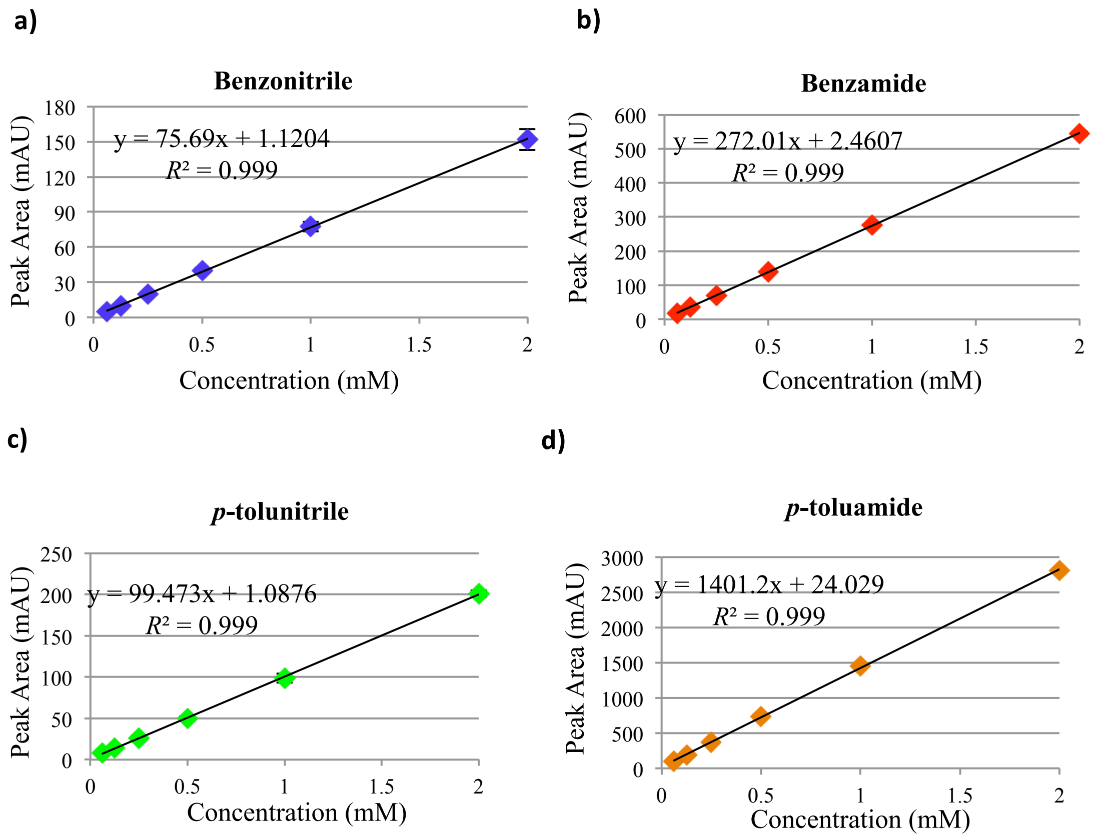

Figure S8. HPLC calibrations for a) benzonitrile b) benzamide c) *p*-tolunitrile and d) *p*-toluamide at 254 nm absorbance. Plots show the mean plus associated standard deviation (SD) error bars from triplicate data.

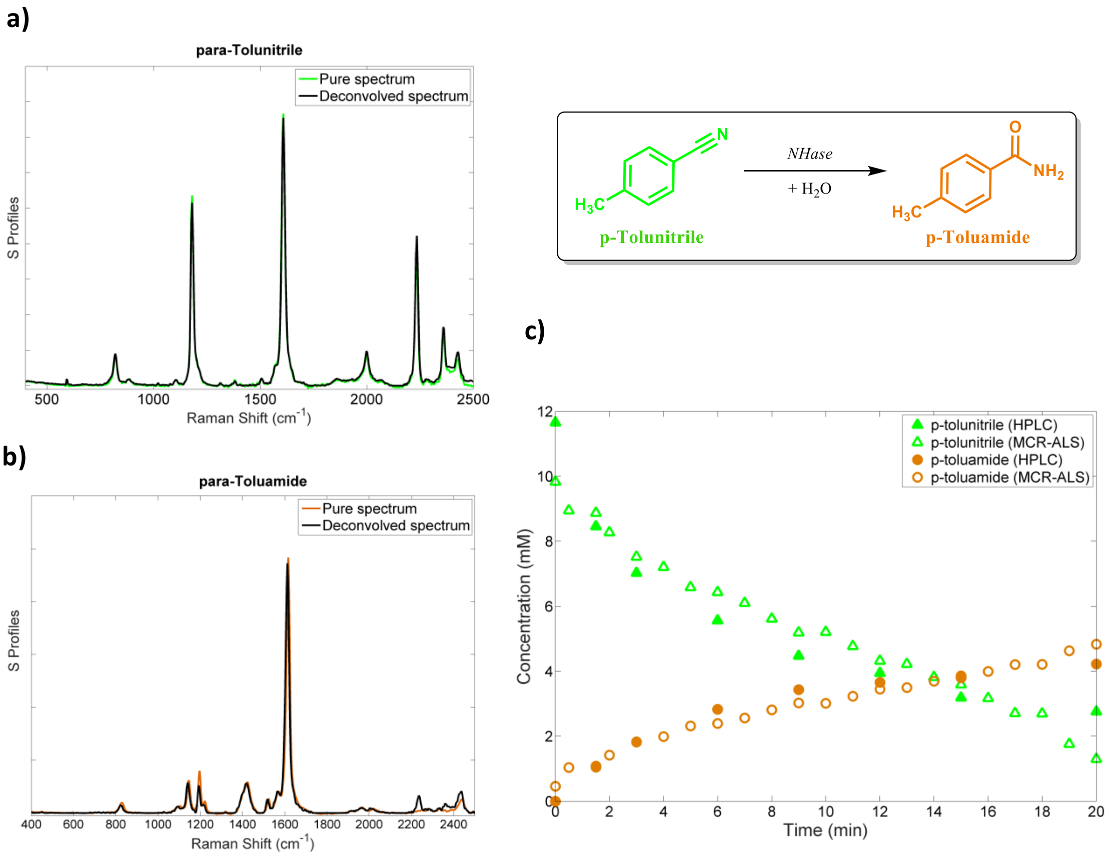

Figure S9. A MCR-ALS model was applied to the UVRR data where it successfully deconvolved spectra into its pure components a) *p*-tolunitrile (green) and b) *p*-toluamide (orange) as shown for biotransformation 2 c) Shows the reaction dynamics from real-time UVRR measurements (denoted by outlined symbols) and off-line HPLC data (denoted by solid symbols) as a function of time for the conversion of *p*-tolunitrile to *p*-toluamide. UVRR spectra were obtained for 20 s with baseline correction, normalisation and smoothing applied (as detailed in ‘Materials and methods: data processing’).

Table S3. The R^2 co-efficients obtained for three replicates for the conversion of *p*-tolunitrile to *p*-toluamide (biotransformation 2).

Replicate	*p*-Tolunitrile R^2	*p*-Toluamide R^2
**1**	0.892	0.860
**2**	0.919	0.943
**3**	0.884	0.938

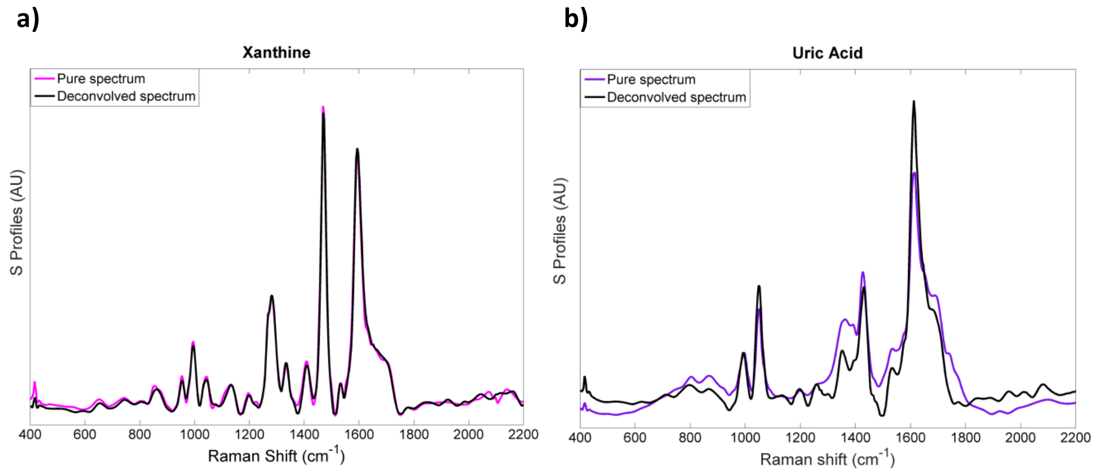

Figure S10 An MCR-ALS model was applied to the UVRR data where it successfully deconvolved spectra into its pure components a) xanthine (substrate) and b) uric acid (product) as shown for biotransformation 3.

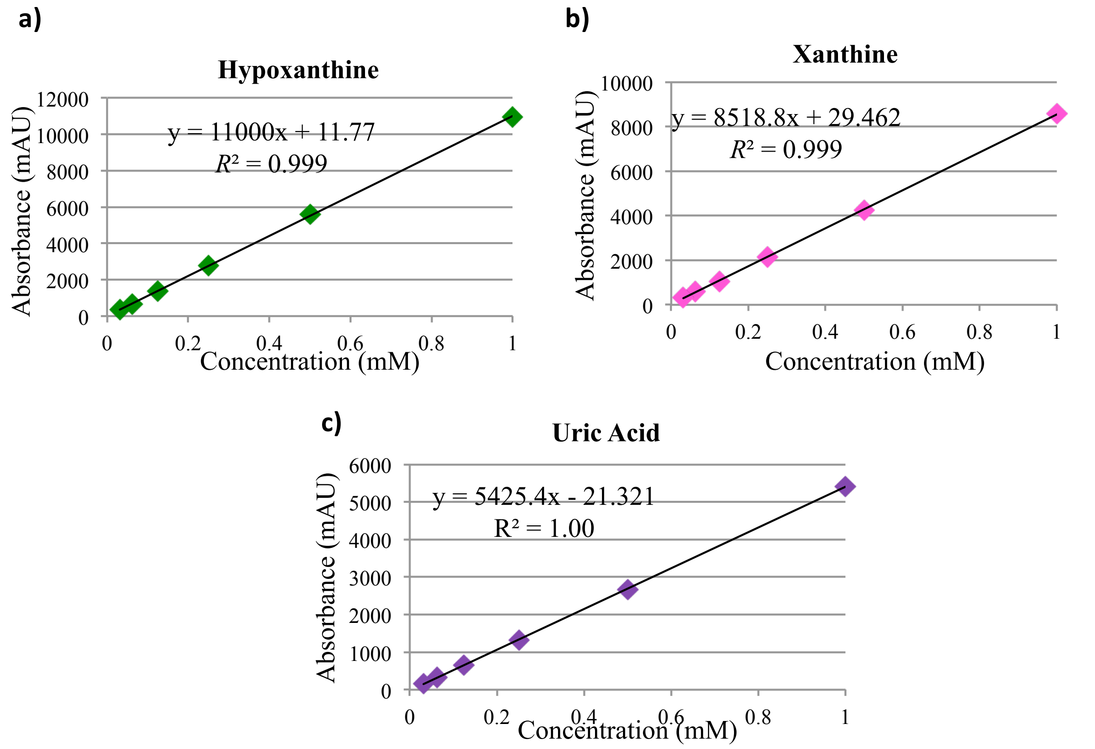

Figure S11. HPLC calibrations for a) hypoxanthine b) xanthine and c) uric acid at 254 nm absorbance. Plots show the mean plus associated standard deviation (SD) error bars from triplicate data.

Table S4. A summary of the R^2 co-efficients across all three replicates for the conversion of hypoxanthine to xanthine to uric acid (biotransformation 4).

**Replicate**	**Hypoxanthine R^2**	**Xanthine R^2**	**Uric Acid R^2**
**1**	0.904	0.171	0.935
**2**	0.736	0.001	0.928
**3**	0.628	0.245	0.765

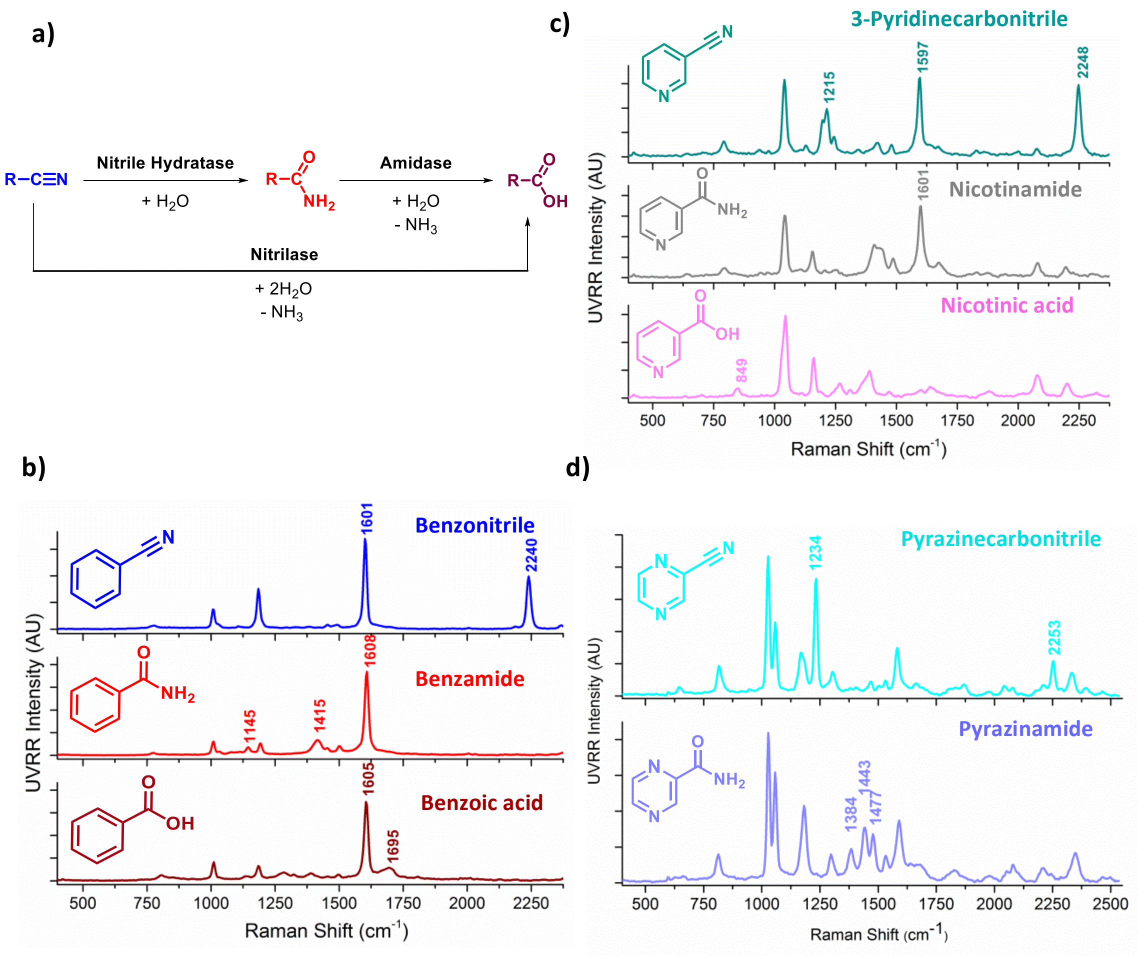

Figure S12. a) Overall schematic illustrating the two known pathways to catalyse the conversion of nitrile containing compounds into their corresponding carboxylic acid, either in a single step (nitrilase) or a multicomponent process (nitrile hydratase and amidase). Plots b-d) show average UVRR spectra (*n*=5) of each analyte: b) pure spectra of benzonitrile, benzamide and benzoic acid (12.5 mM, pH 7.2) c) pure spectra of 3-pyridinecarbonitrile, nicotinamide (vitamin B_3) and nicotinic acid (25 mM, pH 7.2) and d) pure spectra of pyrazinecarbonitrile and pyrazinamide (anti-tuberculosis drug) (25 mM, pH 7.2). Characteristic peaks are annotated. UVRR spectra were obtained for 20 s with baseline correction, normalisation and smoothing applied (as detailed in 'Materials and methods: data processing').

References

[1] a) R. Tauler, *Chemom. Intell. Lab. Syst.* **1995**, *30*, 133-146; b) J. Jaumot, R. Gargallo, A. de Juan, R. Tauler, *Chemom. Intell. Lab. Syst.* **2005**, *76*, 101-110.

[2] a) J. Schwan, S. Ulrich, V. Batori, H. Ehrhardt, S. R. P. Silva, *J. Appl. Phys.* **1996**, *80*, 440-447; b) D. Tallant, T. Friedmann, N. Missert, M. Siegal, J. Sullivan, in *MRS Proceedings, Vol. 498*, Cambridge Univ Press, **1997**, p. 37; c) K. Ishimaru, T. Hata, P. Bronsveld, T. Nishizawa, Y. Imamura, *J. Wood Sci.* **2007**, *53*, 442-448; d) L. Wang, D. Tuschel, S. A. Asher, Proc. *SPIE* **2011**, pp. 80181B-80181B-80186.

[3] a) C. L. Chatterjee, P. Garg, R. M. P. Jaiswal, *Spectrochim. Acta Part A* **1978**, *34*, 943-947; b) X. Gao, J. P. Davies, M. J. Weaver, *J. Phys. Chem.* **1990**, *94*, 6858-6864; c) M. F. Mrozek, S. A. Wasileski, M. J. Weaver, *J. Am. Chem. Soc.* **2001**, *123*, 12817-12825; d) G. D. Fleming, I. Golsio, A. Aracena, F. Celis, L. Vera, R. Koch, M. Campos-Vallette, *Spectrochim. Acta Part A* **2008**, *71*, 1074-1079; e) H. G. Brittain, *Cryst. Growth Des.* **2009**, *9*, 2492-2499.

[4] a) V. R. Kodati, A. T. Tu, J. L. Turumin, *Appl. Spectrosc.* **1990**, *44*, 1134-1136; b) J. Chowdhury, K. M. Mukherjee, T. Misra, *J. Raman Spectrosc.* **2000**, *31*, 427-431; c) V. Krishnakumar, M. Arivazhagan, *Indian J. Pure Appl. Phys.* **2004**, *42*, 411-418; d) M. Arivazhagan, S. Jeyavijayan, *Indian J. Pure Appl. Phys.* **2010**, *48*, 869-874; e) M. Arivazhagan, S. Jeyavijayan, *Spectrochim. Acta Part A* **2011**, *79*, 161-168; f) B. L. Goodall, A. M. Robinson, C. L. Brosseau, *Phys. Chem. Chem. Phys.* **2013**, *15*, 1382-1388.

[5] a) F. Bergmann, S. Dikstein, *J. Am. Chem. Soc.* **1955**, *77*, 691-696; b) M. S. Ascher, A. A. Gottlieb, C. H. Kirkpatrick, *Transfer factor: Basic properties and clinical applications*, Academic Press, **2014**.
